# YTHDF1 promotes the proliferation, migration, and invasion of prostate cancer cells by regulating TRIM44

**DOI:** 10.1007/s13258-021-01175-z

**Published:** 2021-10-22

**Authors:** Weijian Li, Gaohuang Chen, Zhenyu Feng, Baoyi Zhu, Lilin Zhou, Yuying Zhang, Junyan Mai, Chonghe Jiang, Jianwen Zeng

**Affiliations:** 1 Department of Urology, The Sixth Affiliated Hospital of Guangzhou Medical University (Qingyuan People’s Hospital), Qingyuan, 511518 Guangdong China; 2 Department of Urology, The Sixth Affiliated Hospital of Guangzhou Medical University (Qingyuan People’s Hospital), B24 Yinquan Road, Qingcheng, Qingyuan, 511500 Guangdong China

**Keywords:** YTHDF1, TRIM44, Prostate cancer cells, Migration, Invasion

## Abstract

**Background:**

Prostate cancer (PCa) is one of the most common malignancies in men. YTHDF1 may play an important role in promoting PCa progression, but there is no reports to date on YTHDF1 function in PCa.

**Objective:**

This study explored whether YTHDF1 could regulate TRIM44 in PCa cells.

**Methods:**

By querying the TCGA database, we evaluated YTHDF1 expression in PCa, the OS and DFS of YTHDF1, and the correlation between YTHDF1 and TRIM44 in PCa. We constructed vectors to interfere with YTHDF1 expression and overexpress TRIM44 to examine the role of YTHDF1 and TRIM44 in PCa cells. Differentially expressed mRNAs were identified by mRNA sequencing. The levels of YTHDF1, TRIM44, LGR4, SGTA, DDX20, and FZD8 were measured by qRT-PCR and WB was used to determine YTHDF1 and TRIM44 expression. A CCK-8 assay was used to assess cell proliferation. A Transwell chamber assay was used measure cell migration and invasion ability.

**Results:**

YTHDF1 was highly expressed in both Pca tissues and cells. PCa patient prognosis with high YTHDF1 expression was relatively poor. Cell function experiments showed that inhibiting YTHDF1 expression decreased cell proliferation, migration, and invasion. RNA sequencing analysis revealed that YTHDF1 may promote PCa cell proliferation, migration, and invasion by modulating TRIM44 expression. Cell function experiments further verified that YTHDF1 promoted PCa cell proliferation, migration, and invasion by regulating TRIM44.

**Conclusions:**

YTHDF1 enhances PCa cell proliferation, migration, and invasion by regulating TRIM44.

## Introduction

Prostate cancer (PCa) is the most common cancer of solid organs in men and the second most common cause of cancer-related deaths in men (Nguyen-Nielsen and Borre 2016). PCa can present with a variety of clinical manifestations ranging from inert microscopic disease to highly aggressive tumors, including a tendency to metastasize (Lancia et al. 2019). A significant problem for PCa patients is the detection of recurrent disease and the treatment of metastatic cancer (Haberkorn et al. 2016). Most PCa patients receive prostatectomy, radiotherapy, chemotherapy, or hormone therapy, however, the 5-year recurrence rate is approximately 30% (Jemal et al. 2007; Tian et al. 2018). Despite new progress, PCa remains a significant medical problem for men. The high morbidity and mortality of PCa require more work to uncover the underlying mechanisms. In addition, advances in the treatment of PCa require new biomarkers with high accuracy for diagnosis and risk stratification to improve response and outcome.

N6-Methyladenosine (m6A) is the most abundant internal modification of RNA in eukaryotic cells and it regulates mRNA splicing, translation, and stability (Sun et al. 2019). Recent studies indicate that m6A contributes to tumorigenesis through multiple mechanisms (Zhang et al. 2021). Three types of enzymes regulate m6A modification, which include “Writers” (mA methyltransferases including METTL3), “Erasers” (mA demethylases including FTO), and “readers” (mA binding proteins including YTH domain proteins YTHDF1, F2, F3, and heterogeneous nuclear ribonucleoproteins, hnRNPs) (Liang et al. 2020; Meyer and Jaffrey 2017). The “reader” proteins contribute to RNA metabolism through the m6A signal (Xia et al. 2021). YTHDF1 promotes translation efficiency of m6A-modified mRNAs and is associated with a variety of cancers (Wang et al. 2015). YTHDF1 promotes ovarian cancer progression by regulating EIF3C translation (Liu et al. 2020) and plays an important role in other cancers including lung, gastrointestinal, liver, and oral cancers (Nishizawa et al. 2018; Shi et al. 2019; Zhao et al. 2018, 2020). m6A plays an important role in PCa and YTHDF2 is involved in regulating PCa progression (Li et al. 2018). TCGA data indicate that YTHDF1 expression in PCa is higher compared with normal tissues. The prognosis and survival rate of PCa patients with high expression is lower, suggesting that YTHDF1 may play a role in promoting PCa progression. However, there are no studies to date describing a function of YTHDF1 in PCa. Therefore, it is necessary to validate the expression and function of YTHDF1 in PCa.

Tripartite motif containing 44 (TRIM44), an important member of the TRIM family, contributes to a variety of pathological states and is closely associated with malignant tumor etiology and progression (Chen et al. 2021a; Liu et al. 2019; Sato et al. 2021). In addition, TRIM44 gene knockout inhibits PCa cell proliferation and invasion (Tan et al. 2017). These findings suggest that TRIM44 may serve as a prognostic marker and a new therapeutic target for PCa. TCGA data indicate that YTHDF1 and TRIM44 are significantly correlated in PCa and they are both up-regulated. YTHDF1 may promote PCa cell proliferation, migration, and invasion by modulating TRIM44 expression. However, whether YTHDF1 plays a role in PCa cells by regulating TRIM44 remains unclear.

In the present study, we analyzed YTHDF1 expression in PCa and discuss the role of YTHDF1 and TRIM44 in PCa progression. We show that YTHDF1 expression is significantly up-regulated in PCa tissues and cells. Interfering with YTHDF1 expression inhibits cell proliferation, migration, and invasion. Furthermore, YTHDF1 promotes PCa cells proliferation, migration, and invasion by regulating TRIM44. Overall, our study provides a prognostic biomarker and a new therapeutic target for PCa.

## Materials and methods

### Cell culture and transfection

WPMY-1 human normal prostate stromal immutable cells and human PCa cells, PC3, LNCAP, 22RV1, and PC-3 M-IE8 were purchased from the ATCC. The culture conditions for WPMY-1 were DMEM, high glucose, and 5% fetal bovine serum (FBS). PC3 was cultured using F-12 K containing 10% FBS. The culture conditions for LNCAP, 22RV1, and PC-3 M-IE8 consisted of RPMI 1640 containing 10% FBS. To interfere with the expression of YTHDF1, si-YTHDF1 was transfected into the human PCa cell lines, PC3 and LNCap, for 48 h, and then divided into PC3 + si-NC, PC3 + si-YTHDF1, LNCap + si-NC, and LNCap + si-YTHDF1 groups. To investigate whether YTHDF1 plays a role in PCa by regulating TRIM44, we conducted recovery experiments in PC3 cells, which were divided into PC3 + si-NC, PC3 + si-YTHDF1, and PC3 + si-YTHDF1 + TRIM44 groups. si-YTHDF1 (GCUGGCAAAUAUGAAGGUATT) and si-NC (UUCUCCGAACGUGUCACGUTT) constructs were designed and synthesized by GenePharma (Shanghai, China). To overexpress TRIM44, the TRIM44 sequence was cloned into the LV003 vector (Cookgen, Guangzhou, China). Cell transfection was performed using Lipofectamine 2000 (Invitrogen) according to the manufacturer’s instructions.

### Quantitative Real-time PCR (qRT-PCR)

YTHDF1, TRIM44, LGR4, SGTA, DDX20, and FZD8 expression in cells were measured by qRT-PCR. Briefly, total RNA was extracted by the Trizol method, cDNA was reverse-transcribed (Takara, Japan) into cDNA, and the cDNA products were used for qPCR using the SYBR Green Premix kit (Takara, Japan). GAPDH was used as the reference gene and relative gene expression was calculated by the $2^{-\Delta\Delta{\mathrm C}_{\mathrm T}}$ method. The primers used in this study are shown in Table 1.

**Table 1:** The primers used in this study

Primer ID	5′–3′
YTHDF1-F	CAGCACCGATCCCGACATAG
YTHDF1-R	CTGGCTTCCTGAAGACGATGA
TRIM44-F	GCCAGGAAGATAGGCAGCTCAT
TRIM44-R	CTTCAGTCCACCTGAGTCTTTGC
LGR4-F	GGAGCATTTGATGGTAATCCACTC
LGR4-R	CCATGCTTGCACCACGAATGAC
SGTA-F	CGGTAGAAGACAGTGACCTTGC
SGTA-R	TCTGCTGAGTCCTCCTCGGAAG
DDX20-F	AATCAGCGTCTTGATGCTATGGC
DDX20-R	ACAACCAGATTCACCTTCTCAGC
FZD8-F	GCTCTACAACCGCGTCAAGACA
FZD8-R	AAGGTGGACACGAAGCAGAGCA
GAPDH-F	GAGTCAACGGATTTGGTCGT
GAPDH-R	GACAAGCTTCCCGTTCTCAG

### Western blot (WB) analysis

RIPA lysis buffer was used to prepare protein extracts from cells and tissues and the protein was quantitated using the BCA protein assay kit. The samples were mixed with SDS-PAGE loading buffer and heated in a boiling water bath at 100 °C for 5 min. The proteins were transferred to PVDF membranes, blocked for 2 h with 5% skim milk at room temperature, and incubated with YTHDF1 (86,463, CST), TRIM44 (AB236422, ABCAM), and GAPDH (60004-1-LG, Proteintech) primary antibodies for 90 min at room temperature. The membranes were washed three times with TBST and the secondary antibodies were added. The protein bands were developed by chemiluminescence and measured with an Odyssey Infrared Imaging System (Li-Cor Biosciences, Lincoln, NE, USA). GAPDH was used as the internal reference to quantitate relative protein expression levels.

### Cell counting kit 8 (CCK-8) assay

The cells were digested with trypsin. The cell suspension was seeded into 96-well plates at a density of 5 × 10^3^ cells/well. Three replicates were established for each group for treatment according to the experimental groups. The cells were cultured for 24 h. After incubation for a designated period of time, the medium was discarded and replaced with 10 µL CCK-8 working solution and incubated at 37 °C and 5% CO_2_ for another 4 h. The absorbance was measured using an ELX800 microplate reader (BioTek, Winooski, Vermont, USA) at 450 nm. The proliferation ability of cells at 24, 48, and 72 h was determined.

### Cell migration assay

PC3 and LNCaP cell migration were measured by a cell migration assay. Cells (1 × 10^6^/mL) were resuspended in serum-free medium and 100 µL of cell suspension was added to the upper compartment of a Transwell chamber (#33318035, Corning). Complete medium containing 10% FBS was added to the lower chamber and cultured for 48 h. The culture medium was discarded and the chamber was washed twice with PBS. After wiping the upper compartment with a wet cotton swab, they were fixed with 4% paraformaldehyde for 10 min, stained with 0.5% crystal violet for 5 min, and rinsed with water. The cells on the upper and outer surfaces were observed and imaged under a microscope (Olympus, Japan).

### Cell invasion assay

The inner side of the upper chamber of a Transwell chamber was precoated with Matrigel Basement Membrane Matrix (BD Biocoat). The Matrigel was dissolved overnight at 4 °C and then diluted 1:3 with precooled base medium (Matrigel: Medium). Then, 40 µL of Matrigel was added to the pre-cooled Transwell chamber and incubated at 37 °C for 2 h to solidify. The cells were counted with basal medium, adjusted to 1 × 10^6^/mL, and 100 µL and 600 µL complete medium was added to the upper and lower compartments of the Transwell chamber. After incubating at 37 °C for 48 h, the cells in the upper compartment were wiped with a wet cotton swab, fixed with 4 % paraformaldehyde for 10 min, and stained with 0.5% crystal violet for 5 min. A microscope (Olympus, Japan) was used to observe whether the cells entering the bottom cavity through the pore, and images were captured.

### mRNA sequencing and analysis

To further understand the YTHDF1 regulatory mechanism in PCa, transcriptome sequencing analysis was performed on PC3_siRNA_NC and PC3_siRNA_YTHDF1 groups. The RNA sequencing was done by Novogene Bioinformatics Technology Co., Ltd. (Beijing, China). We used an Illumina × 10 sequencing instrument and the average sequencing depth was 100 ×. The R software package was used for quantile normalization to identify differentially expressed mRNAs. The screening criteria were |logFC| > 1 and P < 0.05. Expression patterns of the mRNA were obtained by hierarchical clustering. Gene ontology (GO) and Kyoto Encyclopedia of Genes and Genome (KEGG) analysis were also performed.

### Bioinformatic analysis

By querying the TCGA database (http://starbase.sysu.edu.cn/panGeneDiffExp.php) and (http://gepia.cancer-pku.cn/detail.php?gene=YTHDF1), we analyzed YTHDF1 expression in PCa. The overall survival (OS) and disease-free survival (DFS) of YTHDF1 and the correlation between YTHDF1 and TRIM44 in PCa were determined.

### Statistical analysis

Graphpad 8.0 was used for statistical analysis and the experimental data were expressed as the mean ± SD, which were measured in triplicate. Differences between two groups were analyzed by the Student’s t test. One-way ANOVA was used for comparisons between more than two groups. P < 0.05 was considered statistically significant.

## Results

### YTHDF1 is highly expressed in PCa cells

**Fig. 1:**
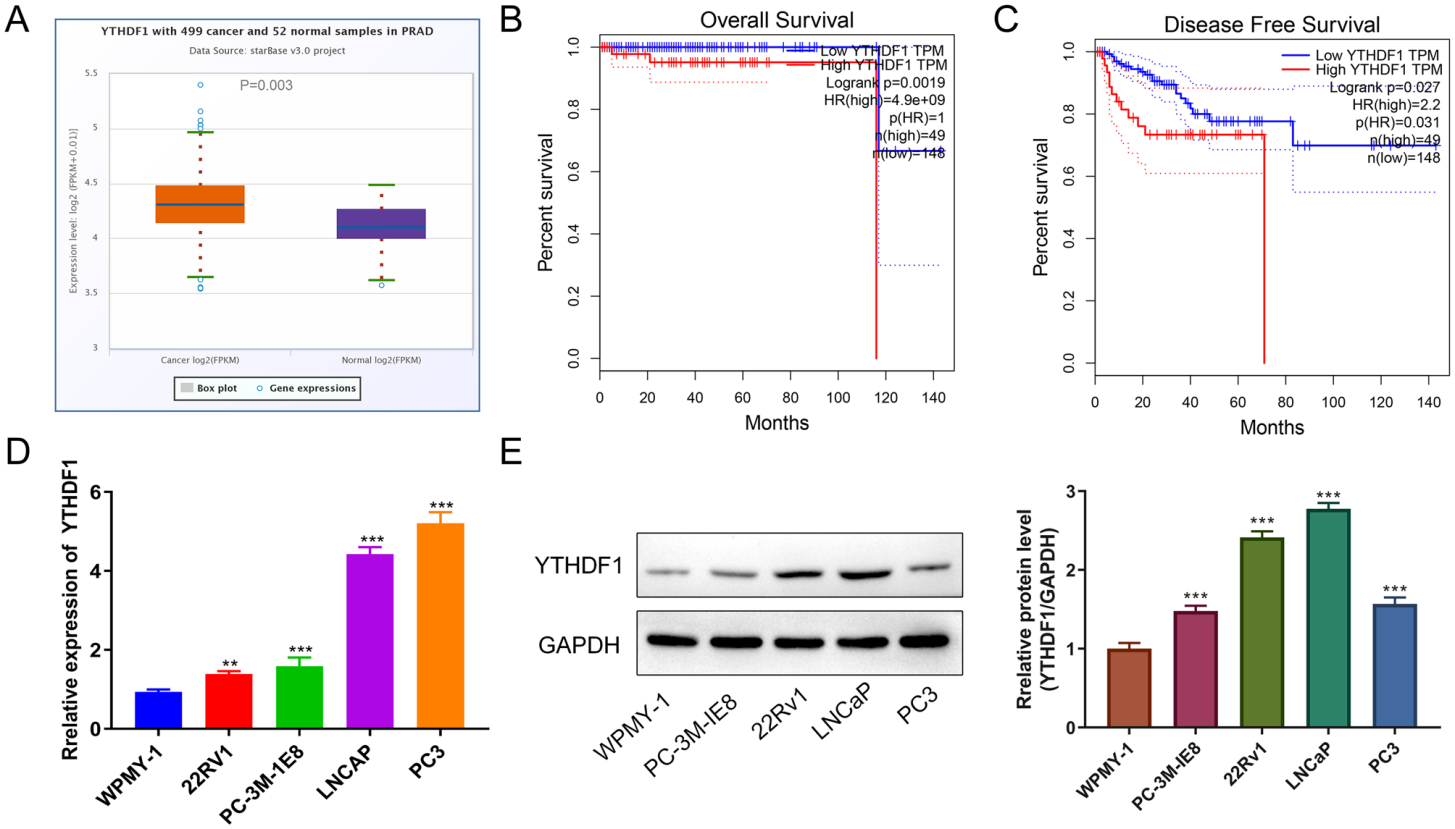
High expression of YTHDF1 in prostate cancer cells. **A** YTHDF1 expression in prostate cancer tissues and paracancerous tissues. **B** The OS of YTHDF1 in PCa. **C** The DFS of YTHDF1 in PCa. **D** qRT-PCR measurement of YTHDF1 expression in WPMY-1 and PCa cells (PC3, LNCap, 22RV1, and PC-3 M-IE8). **E** Western blotting was performed to detect YTHDF1 protein expression in WPMY-1 and prostate cancer cells (PC3, LNCap, 22RV1, and PC-3 M-IE8). *P < 0.05, **P < 0.005, ***P < 0.001

To detect YTHDF1 expression in PCa, we compared 492 PCa tissues (T) and 152 paracancerous tissues (N) by querying the TCGA database and found that YTHDF1 was highly expressed in PCa (P = 0.003, Fig. 1A). Figure 1B C shows the OS and DFS for YTHDF1 in PCa. The results indicate that PCa patients with high YTHDF1 expression have a relatively poor prognosis. To further verify YTHDF1 expression in PCa, qRT-PCR and WB were performed to measure the expression of YTHDF1 in PCa cells and WPMY-1 cells (Fig. 1D, E). The results indicate that YTHDF1 expression in PCa cells was higher compared with that in WPMY-1 cells. These results indicate that YTHDF1 was highly expressed in both PCa tissues and cells and increased expression results in a worse prognosis, which suggests that YTHDF1 may function as a proto-oncogene.

### Suppressing YTHDF1 expression inhibits cell proliferation, migration, and invasion

**Fig. 2:**
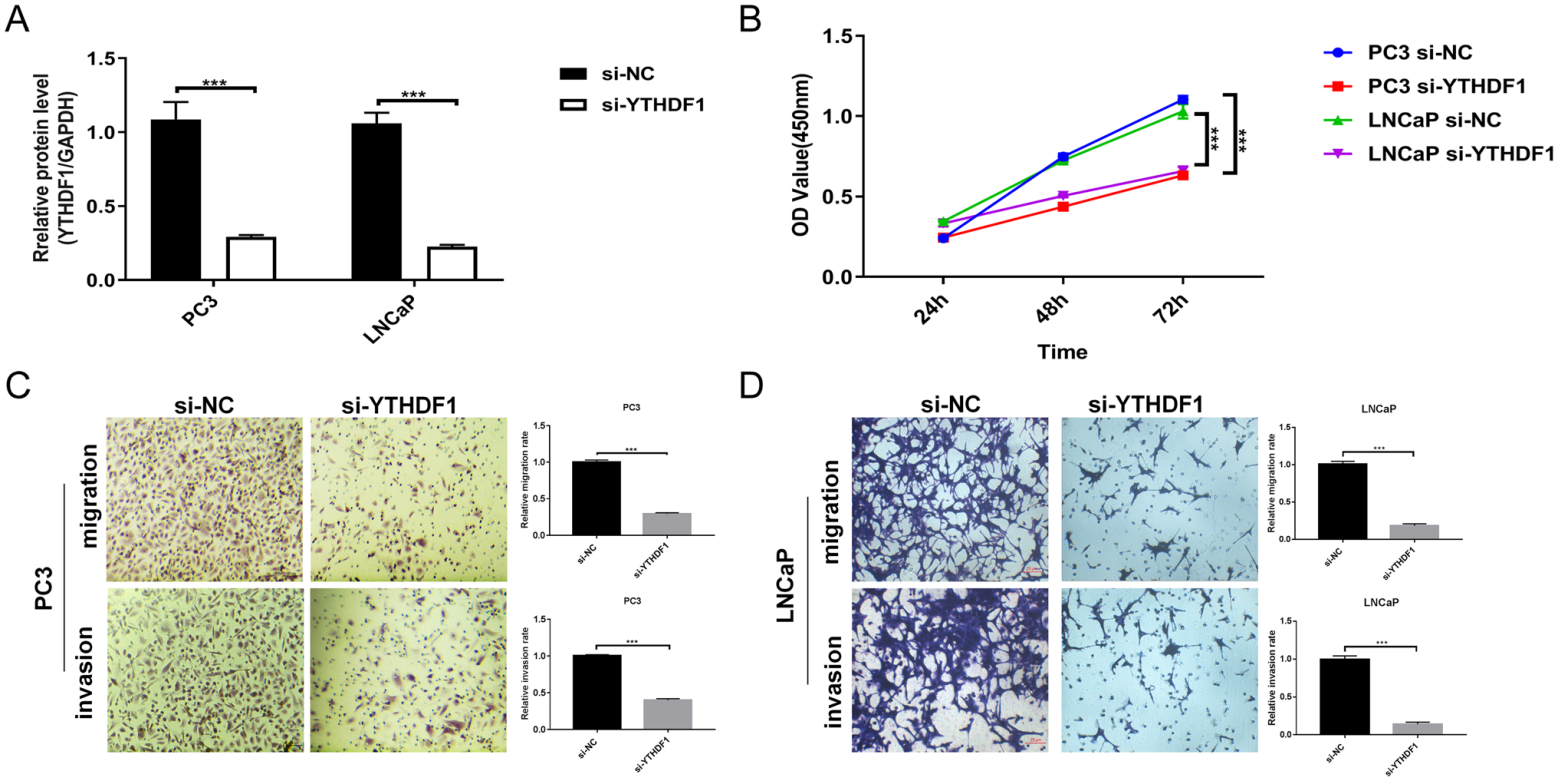
Interfering with YTHDF1 inhibits prostate cancer cell proliferation, migration, and invasion. **A** qRT-PCR measurement of YTHDF1 transfection efficiency. **B** CCK-8 assay was performed to detect PC3 and LNCap cell proliferation at 24, 48, and 72 h. **C** PC3 cell migration and invasion ability after transfection of si-YTHDF1 for 48 h. **D** LNCaP cells migration and invasion ability at 48 h after si-YTHDF1 transfection. Scale bar 50 μm, the magnification is 200 times. *P < 0.05, **P < 0.005, ***P < 0.001

To investigate the effects of YTHDF1 on PCa cells, we transfected si-YTHDF1 interference fragments into PC3 and LNCap cells. As shown in Fig. 2A, YTHDF1 expression was significantly inhibited indicating successful transfection. Cell function experiments revealed that cell proliferation in the si-YTHDF1 group was significantly decreased, whereas the number of migrating and invading cells was reduced compared with the si-NC group (Fig. 2B, D). These results indicate that interfering with the expression of YTHDF1 reduces PCa cells proliferation, migration, and invasion.

### mRNA sequencing analyzing the regulatory mechanism of YTHDF1 in PCa

**Fig. 3:**
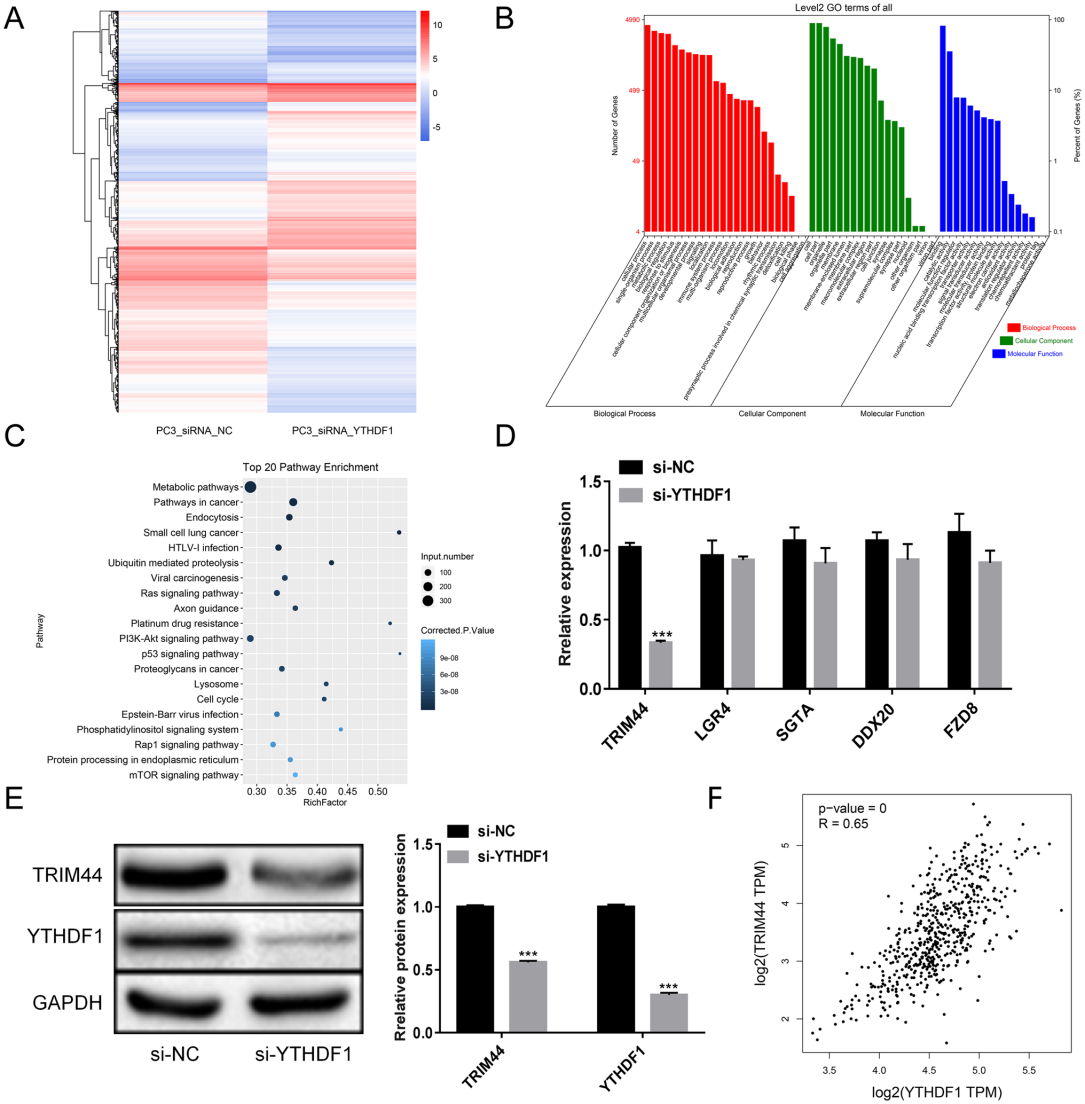
YTHDF1 regulatory mechanism in prostate cancer as analyzed by mRNA sequencing. **A** Analysis of mRNA differential expression in PC3_siRNA_NC and PC3_siRNA_YTHDF1 groups. **B** GO analysis. **C** KEGG analysis. **D** The levels of TRIM44, LGR4, SGTA, DDX20, and FZD8 were measured by qRT-PCR. **E** Western blotting detection of TRIM44 and YTHDF1 levels. **F** The correlation between YTHDF1 and TRIM44. Scale bar 50 μm, the magnification is 200 times. *P < 0.05, **P < 0.005, ***P < 0.001

To further understand the YTHDF1 regulatory mechanism in PCa, we performed mRNA sequencing analysis. The results showed that following YTHDF1 interference, a total of 2992 genes were significantly up-regulated and 2509 genes were significantly down-regulated in PC3 cells (Fig. 3A). GO analysis revealed that the differentially expressed genes were divided into three categories. Among the biological processes, the differentially expressed genes included cellular process, single-organism process, and metabolic regulation. For the cellular component classification, the differentially expressed genes were mainly divided into the cell, cell part, organelle part, and membrane. For the classification of molecular function, differentially expressed genes were mainly divided into the binding, catalytic activity, and molecular function regulator (Fig. 3B). Using KEGG, we analyzed the top 20 signaling pathways and identified PI3K-Akt pathway, which plays a major role in regulating cell proliferation, growth, differentiation, and apoptosis (Fig. 3C). We selected five genes (TRIM44, LGR4, SGTA, DDX20, and FZD8) that have been reported previously in PCa and are considered significant differentially expressed genes. qRT-PCR and WB revealed TRIM44 mRNA and protein expression were significantly down-regulated in the si-YTHDF1group (Fig. 3D, E). The TCGA database indicated that YTHDF1 and TRIM44 are significantly correlated in PCa, and they were both up-regulated (Fig. 3F). This suggests that YTHDF1 may promote PCa cells proliferation, migration, and invasion by modulating TRIM44 expression.

### YTHDF1 regulates TRIM44 to promote PCa cells proliferation, migration, and invasion

**Fig. 4:**
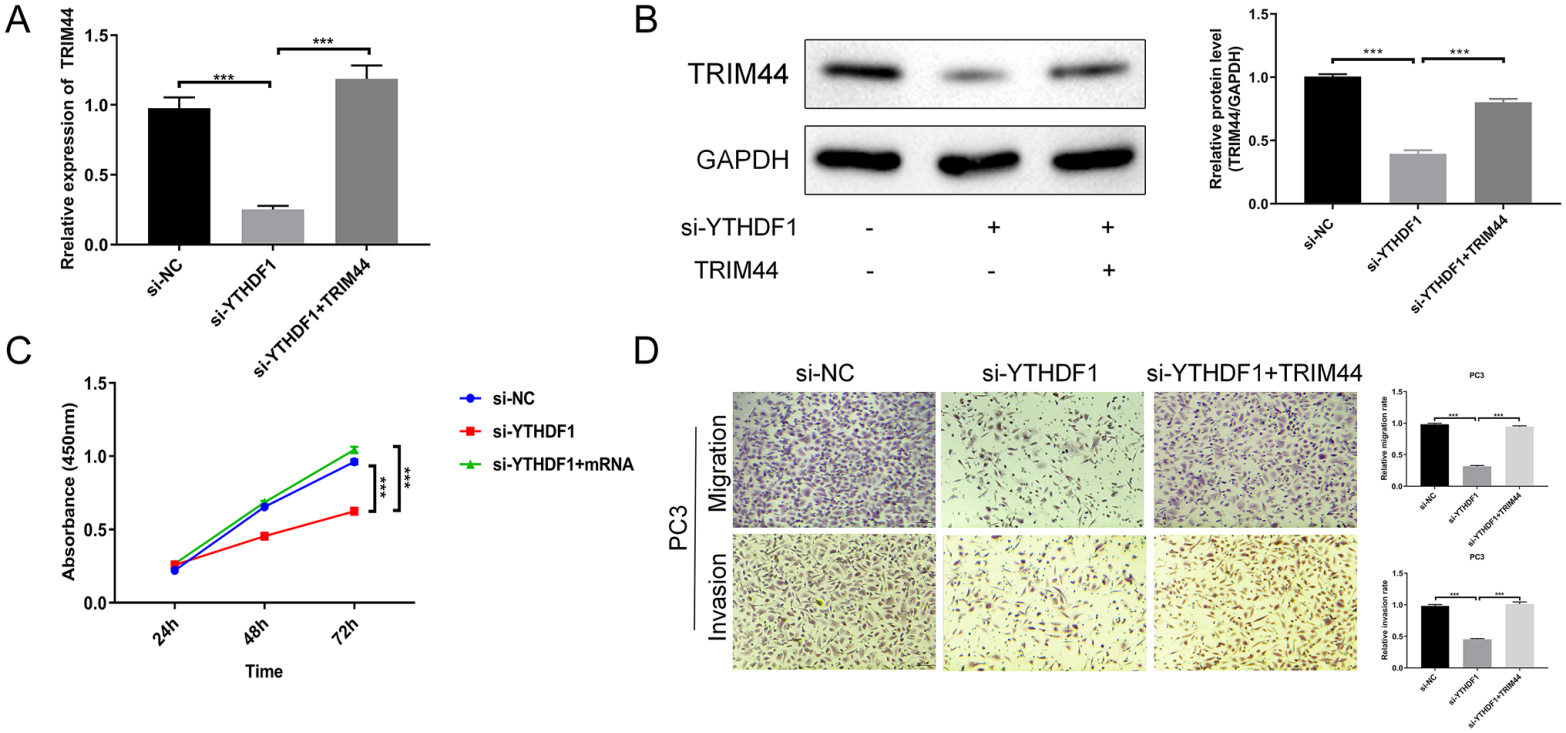
YTHDF1 regulates TRIM44 to promote prostate cancer cells proliferation, migration, and invasion. **A** qRT-PCR determination of TRIM44 transfection efficiency. **B** Western blotting detection of TRIM44 protein levels. **C** CCK-8 assay was performed to detect PC3 cell proliferation at 24, 48, and 72 h. **D** PC3 cell migration and invasion ability after transfection with TRIM44 for 48 h. Scale bar 50 μm, the magnification is 200 times. *P < 0.05, **P < 0.005, ***P < 0.001

To explore whether YTHDF1 functions by regulating TRIM44 in PCa cells, we performed recovery experiments in PC3 cells. As shown in Fig. 4A, after overexpression of TRIM44 in PC3 cells, the level of TRIM44 was up-regulated, indicating a successful transfection. To further verify the expression of TRIM44, we performed WB experiments. The results indicated that after overexpressing TRIM44 in PC3 cells, the expression level of TRIM44 protein was significantly up-regulated (Fig. 4B). Cell function experiments showed that cell proliferation, migration, and invasion ability were restored after overexpressing TRIM44 in PC3 cells (Fig. 4C, D). This suggests that YTHDF1 promotes PCa cells proliferation, migration, and invasion by regulating TRIM44.

## Discussion

PCa is regarded as a common cancer with a high mortality rate in men (Jain and Sapra 2021). Current markers used for PCa detection, such as prostate-specific antigen screening, lead to significant overtreatment (Loeb and Giri 2021). As a reversible epigenetic modification, m6A is associated with the occurrence, metastasis, and drug resistance of tumors (Huang et al. 2020). YTHDF1 plays an essential role in a variety of cancers as a core factor of RNA methylation modification (Yarmishyn et al. 2020; Zhang et al. 2020). However, its specific mechanism in PCa has not been defined. In the present study, we analyzed YTHDF1 expression in PCa by querying the TCGA database and found that YTHDF1 was highly expressed in PCa tissues. PCa patients with high expression of YTHDF1 had a relatively poor prognosis, indicating that it may function as a proto-oncogene.

YTHDF1 is a “reader” of m6A-modified mRNA (Wang et al. 2015). The change in m6A levels may be involved in PCa occurrence and development (Wu et al. 2021). Previous studies have reported that YTHDF1 promotes the occurrence and metastasis of gastric cancer in an m6A-dependent manner by promoting the translation of USP14 and it may represent a potential target for gastric cancer treatment (Chen et al. 2021c). YTHDF1 aggravates cervical cancer progression through m6A-mediated RANBP2 upregulation (Wang et al. 2021). Overexpression of YTHDF1 can reduce the sensitivity of colon cancer cells to cisplatin (Chen et al. 2021b). Consistent with previous studies, we found that YTHDF1 was highly expressed in prostate cancer cells and reduced expression of YTHDF1 inhibited PCa cell proliferation, migration, and invasion, suggesting that YTHDF1 may represent a target for PCa treatment.

The most common cause of death from PCa is metastasis (Krishna and Bergan 2014). TRIM44 plays an essential role in cancer progression. Overexpression of TRIM44 promotes cell proliferation and enhances hepatocellular carcinoma cell invasion and migration (Zhu et al. 2016). Knocking down TRIM44 inhibits the proliferation of renal cell carcinoma (Yamada et al. 2020). In PCa, TRIM44 gene knockout can inhibit PCa cell proliferation and invasion (Tan et al. 2017). Our results showed that YTHDF1 and TRIM44 were significantly correlated in PCa and they were both up-regulated. This suggests that YTHDF1 may promote PCa cell proliferation, migration, and invasion by modulating the expression of TRIM44. Therefore, we performed recovery experiments to confirm that YTHDF1 promotes PCa cell proliferation, migration, and invasion by regulating TRIM44. This study provides new insight into PCa etiology and progression. However, there are still limitations to our study. It is a basic in vitro study, which provides only a preliminary discussion on the molecular mechanism of YTHDF1 and TRIM44. Therefore, further studies in animals will be needed.

## Conclusions

This study provided evidence that YTHDF1 promotes PCa cells proliferation, migration, and invasion by regulating TRIM44. Defining the molecular mechanism through which YTHDF1 regulates TRIM44 may be helpful to identify potential targets for PCa treatment to control its growth and metastasis.
